# Supplementary material for: Haploinsufficiency of Adenomatous Polyposis Coli Coupled with Kirsten Rat Sarcoma Viral Oncogene Homologue Activation and P53 Loss Provokes High-Grade Glioblastoma Formation in Mice
Source: Cancers (Basel). 2024 Mar 4;16(5):1046. doi: 10.3390/cancers16051046 (PMC10930734; doi:10.3390/cancers16051046)
Supplement: Supplementary file 1 [file cancers-16-01046-s001.zip › Mouse behavior test Suppl Table S3.pdf]

Supplementary Table S3: The representative results of our behavioral testing for GBM mice.

Mice No.	Genotype	Age (wks)	Sex	Frailty	Paralysis (Crippled)	Head tilt	Teetering	Epilepsy	Headstand circling
568	GFAP.Kras.APC(L/+).P53(LL)	7W	F	X	V(left forelimb weakness)	V(turn right side)	X	X	V
859	GFAP.Kras.APC(L+).P53(LL)	7W	F	V	V(left forelimb weakness)	V(turn right side)	X	X	V
461	GFAP.Kras.APC(L+).P53(LL)	7.5W	M	V	V(both limb muscles weakness)	X(enlarged domed skull)	V	X	X (limb weakness)
723	GFAP.Kras.APC(L+).P53(LL)	8.2W	F	V	V (hindlimb weakness)	X(enlarged domed skull)	V	X	X (limb weakness)
720	GFAP.Kras.APC(L+).P53(LL)	8.2W	F	V	V (hindlimb weakness)	X	V	X	X (limb weakness)
251	GFAP.Kras.APC(L+).P53(LL)	7.4W	F	V	V(both limb muscles weakness)	X(enlarged domed skull)	V	X	X
239	GFAP.Kras.APC(L+).P53(LL)	7.4W	F	V	V (both limb muscles weakness)	X	X	X	X
728	GFAP.Kras.APC(L+).P53(LL)	8.2W	M	X	V (both limb muscles weakness)	X(enlarged domed skull)	X	X	X
965	GFAP.Kras.APC(L+).P53(LL)	7W	F	V	V (both limb muscles weakness)	X(enlarged domed skull)	X	X	X
727	GFAPKras.APC(L+).P53(LL)	7.57W	F	V	V((hindlimb weakness))	X(enlarged domed skull)	V	X	X((limb weakness)

861	GFAP.Kras.P53(LL)	9.5W	F	V	V(left forelimb weakness)	V(turn right side)	V(weak grip)	X	X
493	GFAP.Kras.P53(LL)	8.7W	M	V	V (both limb muscles weakness)	X	V(weak grip)	X	X
660	GFAP.Kras.P53(LL)	9.2W	F	V	V(left forelimb weakness)	X	V(weak grip)	X	X
440	GFAP.Kras.P53(LL)	9.4W	F	V	V (hindlimb weakness)	X	V(weak grip)	X	X
911	GFAP.Kras.P53(LL)	9.2W	F	V	V (hindlimb weakness)	X	V(weak grip)	X	X
84	GFAP.Kras.P53(LL)	9.5W	F	V	V (hindlimb weakness)	X	V (weak grip)	X	X
90	GFAP.Kras.P53(LL)	9W	F	V	V (forelimb weakness)	X	V(weak grip)	X	X
112	GFAP.Kras.P53(L+)	18W	M	X	X (forelimb weakness)	X	X	X	X
127	GFAP.APC(L+).P53(LL)	20W	M	X	X	X	X	X	X
129	GFAP.APC(L+).P53(LL)	20W	M	X	X	X	X	X	X
258	GFAP.APC(LL).P53(LL)	3.43W	F	V	V	V	X	X	X
A018	GFAP.APC(LL).P53(LL)	2.71W	F	V	V (hindlimb weakness)	X(dent in skull)	V	X	X (limb weakness)
